# Modulation of the Blood–Brain Barrier by Sigma-1R Activation

**DOI:** 10.3390/ijms25105147

**Published:** 2024-05-09

**Authors:** Eugen Brailoiu, Jeffrey L. Barr, Hailey N. Wittorf, Saadet Inan, Ellen M. Unterwald, Gabriela Cristina Brailoiu

**Affiliations:** 1 Center for Substance Abuse Research, Lewis Katz School of Medicine at Temple University, Philadelphia, PA 19140, USA; eugen.brailoiu@temple.edu (E.B.); jeffrey.barr@sanfordhealth.org (J.L.B.); saadet.inan@temple.edu (S.I.); 2 Department of Neural Sciences, Lewis Katz School of Medicine at Temple University, Philadelphia, PA 19140, USA; 3 Department of Pharmaceutical Sciences, Jefferson College of Pharmacy, Thomas Jefferson University, Philadelphia, PA 19107, USA; hailey.wittorf@jefferson.edu

**Keywords:** Sigma-1R, BBB, rat brain microvascular endothelial cells, ECIS

## Abstract

Sigma non-opioid intracellular receptor 1 (Sigma-1R) is an intracellular chaperone protein residing on the endoplasmic reticulum at the mitochondrial-associated membrane (MAM) region. Sigma-1R is abundant in the brain and is involved in several physiological processes as well as in various disease states. The role of Sigma-1R at the blood–brain barrier (BBB) is incompletely characterized. In this study, the effect of Sigma-1R activation was investigated in vitro on rat brain microvascular endothelial cells (RBMVEC), an important component of the blood–brain barrier (BBB), and in vivo on BBB permeability in rats. The Sigma-1R agonist PRE-084 produced a dose-dependent increase in mitochondrial calcium, and mitochondrial and cytosolic reactive oxygen species (ROS) in RBMVEC. PRE-084 decreased the electrical resistance of the RBMVEC monolayer, measured with the electric cell-substrate impedance sensing (ECIS) method, indicating barrier disruption. These effects were reduced by pretreatment with Sigma-1R antagonists, BD 1047 and NE 100. In vivo assessment of BBB permeability in rats indicates that PRE-084 produced a dose-dependent increase in brain extravasation of Evans Blue and sodium fluorescein brain; the effect was reduced by the Sigma-1R antagonists. Immunocytochemistry studies indicate that PRE-084 produced a disruption of tight and adherens junctions and actin cytoskeleton. The brain microcirculation was directly visualized in vivo in the prefrontal cortex of awake rats with a miniature integrated fluorescence microscope (aka, miniscope; Doric Lenses Inc.). Miniscope studies indicate that PRE-084 increased sodium fluorescein extravasation in vivo. Taken together, these results indicate that Sigma-1R activation promoted oxidative stress and increased BBB permeability.

## 1. Introduction

Sigma non-opioid intracellular receptor 1 (Sigma-1R) [1] is an intracellular protein located on the endoplasmic reticulum (ER), clustered at the mitochondria-associated ER membrane (MAM) domains [2,3,4]. In response to agonist stimulation, Sigma-1R dissociates from the complex with other proteins and translocates to the cell membrane, where it regulates the function of receptors and channels, or to the nucleus where it regulates gene transcription [2,5]. Sigma-1R activation potentiates Ca^2+^ mobilization induced by inositol 1,4,5-triphosphate (IP_3_)- generating mediators, modulates Ca^2+^ influx into mitochondria [6,7,8], and inhibits store-operated Ca^2+^ entry (SOCE) [9,10].

Sigma-1R regulates several processes, such as neurotransmitter release, neuritogenesis, neural plasticity, long-term potentiation, and apoptosis [11,12]. Sigma-1Rs bind diverse ligands, including antidepressants (e.g., fluoxetine), antipsychotics (e.g., haloperidol), and drugs of abuse (e.g., cocaine and methamphetamine) [13,14,15,16]. Sigma-1R ligands have been proposed as pharmacological tools in the treatment of depression, anxiety, amnesia, schizophrenia, Alzheimer’s disease, Parkinson’s disease, pain, and addiction [12,17,18].

Sigma-1Rs are abundant in the brain [19,20,21] and expressed in brain microvascular endothelial cells [9,22]. There is limited and controversial information regarding the effect of Sigma-1R activation on blood–brain barrier (BBB) permeability. Drugs of abuse, such as cocaine and methamphetamine, bind Sigma-1R [23] and impair BBB function [24,25,26,27]. We have demonstrated the increase in BBB permeability by cocaine by direct visualization with miniaturized fluorescence microscopy [28]. However, chronic administration of the Sigma-1R agonist, PRE-084, attenuated BBB dysfunction in mouse models of vascular dementia [22] and Alzheimer’s disease [29].

Here, we investigated the effect of Sigma-1R activation on mitochondrial Ca^2+^ and reactive oxygen species in vitro using rat brain microvascular endothelial cells (RBMVEC) and in vivo, on BBB permeability in rats using Evans Blue and sodium fluorescein extravasation methods and live imaging with a miniscope in awake rats.

## 2. Results

### 2.1. PRE-084 Increases Mitochondrial Ca^2+^ in RBMVEC

**Figure 1:**
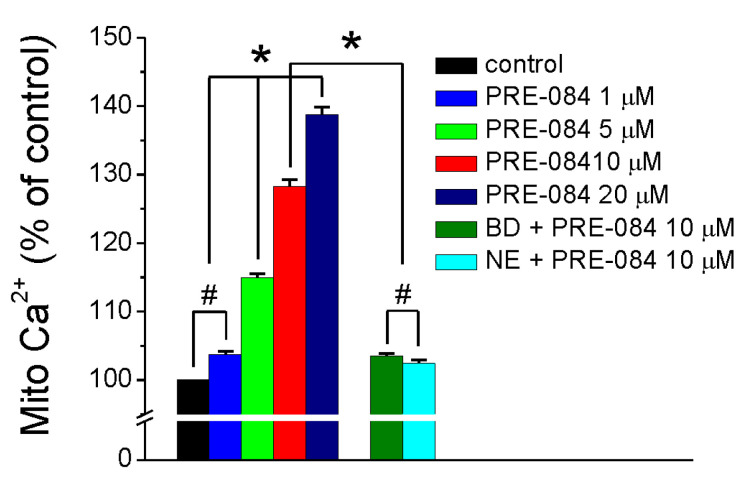
PRE-084 increases mitochondrial Ca^2+^ in RBMVEC. PRE-084 (5, 10, 20 µM) produced a dose-dependent increase in mitochondrial Ca^2+^ (Mito Ca^2+^) in RBMVEC loaded with the mitochondrial Ca^2+^ indicator, Rhod-2 AM. Pretreatment with Sigma-1R antagonists BD 1047 (25 µM) or NE 100 (5 µM) markedly decreased the effect of PRE-084 (10 µM); n = 40–55 cells/treatment group. * *p* < 0.05; #, not statistically significant.

In RBMVEC loaded with Rhod-2 AM, PRE-084 (5 µM, 10 µM, and 20 µM), Sigma-1R selective agonist [30], produced a dose-dependent increase in Rhod-2 AM fluorescence intensity, indicative of the increase in mitochondrial Ca^2+^ (Mito Ca^2+^, Figure 1). PRE-084 (1 µM) did not produce a statistically significant increase in Rhod-2 AM fluorescence as compared to the control (Figure 1). Pretreatment with BD 1047 (25 µM) or NE 100 (5 µM), Sigma-1R antagonists [31,32], while not eliciting a change in Rhod-2 AM fluorescence markedly decreased the effect of PRE-084 (10 µM) (Figure 1), indicating that the effect was mediated via Sigma-1R.

### 2.2. PRE-084 Increases Mitochondrial Superoxide in RBMVEC

**Figure 2:**
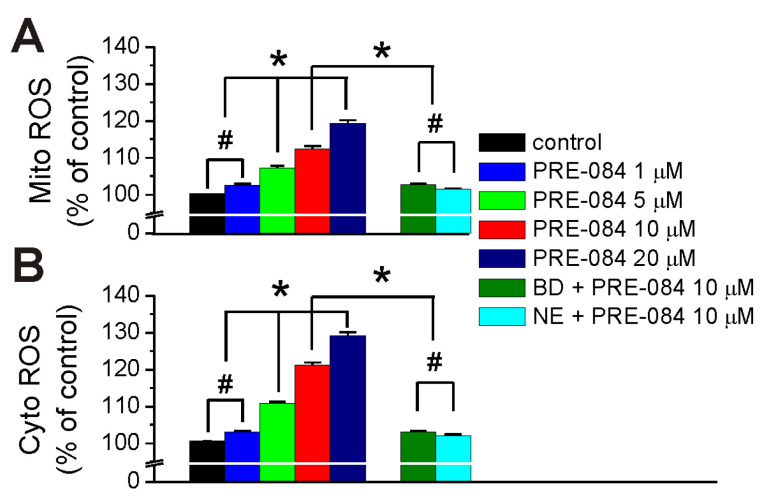
PRE-084 increases mitochondrial superoxide and cytosolic reactive oxygen species in RBMVEC. (**A**), PRE-084 (5 µM, 10 µM, and 20 µM) produced a concentration-dependent increase in MitoSOX fluorescence, indicating an increase in mitochondrial superoxide (Mito ROS). Pretreatment with Sigma-1R antagonists, BD 1047 (25 µM) or NE 100 (5 µM) prevented the increase in Mito ROS by PRE-084 (10 µM). (**B**), PRE-084 (5 µM, 10 µM, and 20 µM) produced a concentration-dependent increase in CM-H_2_-DCFDA fluorescence intensity, indicating an increase in cytosolic ROS (Cyto ROS) production. The response to PRE-084 (10 µM) was abolished by pretreatment with BD 1047 (25 µM) or NE 100 (5 µM); n = 43–55 cells/treatment group. * *p* < 0.05; #, not statistically significant.

In other series of experiments, RBMVEC were loaded with MitoSOX Red, a dye that selectively targets mitochondria and is an indicator for mitochondrial superoxide production [33]. PRE-084 (5 µM, 10 µM, and 20 µM) produced a concentration-dependent increase in MitoSOX fluorescence indicative of an increase in mitochondrial superoxide (Mito ROS, Figure 2A). PRE-084 (1 µM) did not produce an increase in MitoSOX fluorescence different from the control (Figure 2A). Pretreatment with BD 1047 (25 µM) or NE 100 (5 µM) did not increase MitoSOX fluorescence; it prevented the increase in MitoSOX fluorescence by PRE-084 (10 µM) (Figure 2A).

### 2.3. PRE-084 Increases Cytosolic Reactive Oxygen Species in RBMVEC

In RBMVEC loaded with CM-H_2_-DCFDA, an indicator for cytosolic reactive oxygen species (ROS), PRE-084 (5 µM, 10 µM, and 20 µM) produced a concentration-dependent increase in the CM-H_2_-DCFDA fluorescence intensity, suggesting an increase in cytosolic ROS (Cyto ROS) production (Figure 2B). The response to PRE-084 (10 µM) was abolished by pretreatment with BD 1047 (25 µM) or NE 100 (5 µM) (Figure 2B).

### 2.4. PRE-084 Disrupts Endothelial Barrier Function In Vitro

**Figure 3:**
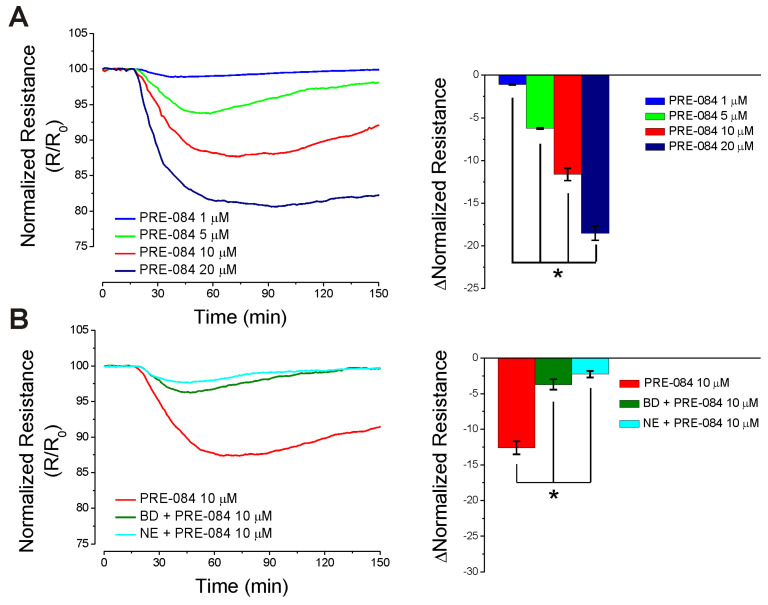
PRE-084 decreases the electrical resistance of the RBMVEC monolayer. (**A**), Examples of decreases in RBMVEC normalized resistance produced by PRE-084 (1–20 µM) determined with ECIS over a period of 150 min (left panel) and comparison of the decrease in amplitude of normalized resistance (right panel). (**B**), Examples of the decrease in normalized resistance produced by PRE-084 (10 µM) in the absence and presence of Sigma-1R antagonists, BD 1047 (25 µM) and NE 100 (5 µM) (left panel), and comparison of the amplitude of decrease in normalized resistance in each condition (right panel); n = 6 experiments/treatment group. * *p* < 0.05.

Impedance measurements with the ECIS method were carried out in RBMVEC monolayers grown on gold electrodes (8W10E+ arrays). Treatment of confluent RBMVEC monolayers with PRE-084 (5 µM, 10 µM, and 20 µM) produced a fast and transient, concentration-dependent decrease in electrical resistance measured at 4000 Hz, indicating a transient disruption of the endothelial barrier function. PRE-084 (1 µM) did not produce a significant change in electrical resistance. Examples of changes in normalized resistance (R/R_0_) produced by PRE-084 (1–20 µM) and a comparison of the decrease in normalized resistance are illustrated in Figure 3A. Pretreatment of RBMVEC on ECIS arrays with BD 1047 (25 µM) or NE 100 (5 µM) produced a marked decrease in the response to PRE-084 (10 µM) (Figure 3B).

### 2.5. PRE-084 Alters Tight and Adherens Junctions and Actin Cytoskeleton in RBMVEC

**Figure 4:**
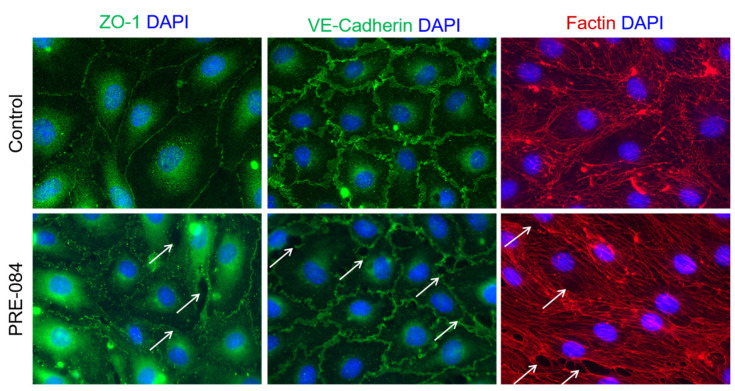
PRE-084 produced a disruption in tight and adherens junction proteins and F-actin in RBMVEC. Distribution of tight junction accessory protein ZO-1, adherens junction protein VE-cadherin, and cytoskeleton component F-actin in the control (untreated) RBMVEC (top panels) and RBMVEC treated with PRE-084 (10 µM, 30 min, bottom panels). Nuclei are stained with DAPI. Treatment with PRE-084 produced a disruption in ZO-1 and VE-cadherin, reorganization of F-actin, and the formation of intercellular gaps (arrows). Microscope objective 63X oil.

Immunocytochemistry studies were carried out to examine the distribution of ZO-1, a tight junction accessory protein, VE-cadherin, a component of adherens junction, and of F-actin cytoskeleton in RBMVEC after treatment with PRE-084. In the control, untreated, RBMVEC, ZO-1, and VE-cadherin immunostaining were visualized as fine structures bordering the cells and F-actin cytoskeleton as intracellular filaments throughout the cells (Figure 4 top panels). Treatment of RBMVEC with PRE-084 (10 μM, 30 min) produced a disruption in ZO-1 and VE-cadherin immunoreactivity and a reorganization of F-actin stress fibers leading to the formation of intercellular gaps, indicated with arrows (Figure 4 bottom panels).

### 2.6. PRE-084 Increases the BBB Permeability In Vivo

**Figure 5:**
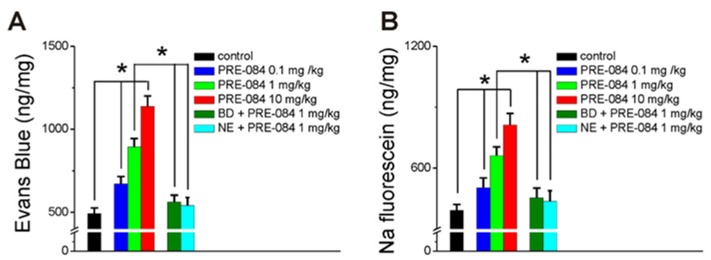
PRE-084 increases the BBB permeability in rats assessed with Evans Blue and sodium fluorescein. (**A**), PRE-084 (0.1 mg/kg, 1 mg/kg, and 10 mg/kg, i.p.) produced a dose-dependent increase in Evans Blue extravasation in the rat brain; BD 1047 (10 mg/kg) and NE 100 (1 mg/kg) markedly reduced the effect of PRE-084. (**B**) PRE-084 (0.1 mg/kg, 1 mg/kg, and 10 mg/kg, i.p.) produced a dose-dependent increase in Na fluorescein extravasation in the rat brain, that was prevented by pretreatment with the Sigma-1R antagonists, BD 1047 and NE 100; n = 6 rats/treatment group; * *p* < 0.05.

The BBB permeability was assessed in rats with Evans Blue and sodium (Na) fluorescein, two widely used tracers [34]. Systemic administration of PRE-084 (0.1 mg/kg, 1 mg/kg, and 10 mg/kg, i.p.) produced a dose-dependent increase in the brain concentration of Evans Blue and Na fluorescein (n = 6 rats) (Figure 5). In control (vehicle-treated) rats, brain concentrations of Evans Blue or Na fluorescein were similar to previous reports [35,36]. Pretreatment with BD 1047 (10 mg/kg) or NE 100 (1 mg/kg) before PRE-084 (1 mg/kg) significantly attenuated the increase in the brain concentration of Evans Blue and Na fluorescein produced by PRE-084 to levels similar to control levels (n = 6 rats) (Figure 5).

### 2.7. PRE-084 Increases Sodium Fluorescein Brain Extravasation In Vivo Visualized with Miniscope

**Figure 6:**
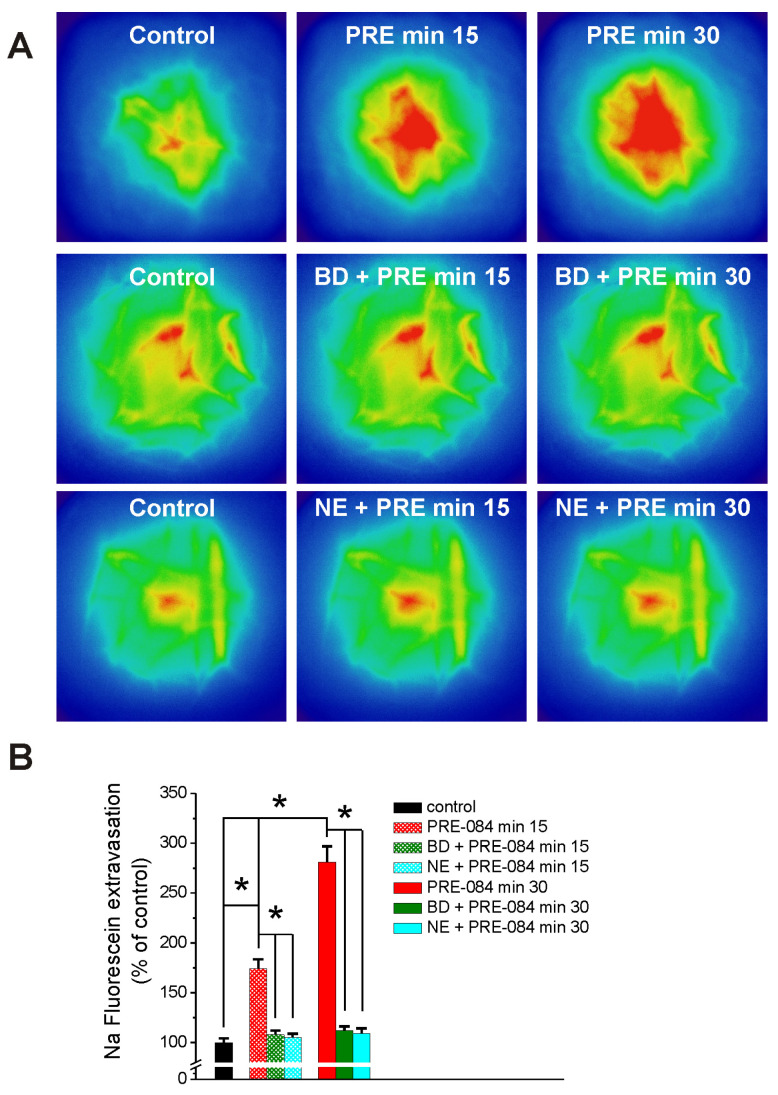
PRE-084 increases sodium fluorescein extravasation in rat brain microvessels visualized with a miniscope. (**A**), Pseudocolor images (GRIN lens, Doric Lenses) illustrating Na fluorescein extravasation in rat prefrontal cortex microvessels before (control), 15, and 30 min after injection of PRE-084 (1 mg/kg) in the absence or presence of BD 1047 (10 mg/kg) and NE 100 (1 mg/kg). (**B**), Comparison of Na fluorescein extravasation in brain microvessels in each condition. n = 5 rats/treatment group; * *p* < 0.05.

Brain microcirculation in the prefrontal cortex was visualized with the miniscope following sodium fluorescein administration in awake, freely moving rats before (control) and 15 and 30 min after systemic administration of PRE-084 (1 mg/kg, i.p.). PRE-084 (1 mg/kg) increased sodium fluorescein extravasation to 174 ± 9.4% and 281 ± 15.9% of control at 15 and 30 min, respectively, after injection. Pretreatment with BD 1047 (10 mg/kg) and NE 100 (1 mg/kg) reduced the Na fluorescein extravasation produced by PRE-084. Pseudocolor images of fluorescence produced by Na fluorescein in the brain microvessels are illustrated in Figure 6A and a comparison of Na fluorescein extravasation in each condition compared to the baseline (before PRE-084) is illustrated in Figure 6B.

**Figure 7:**
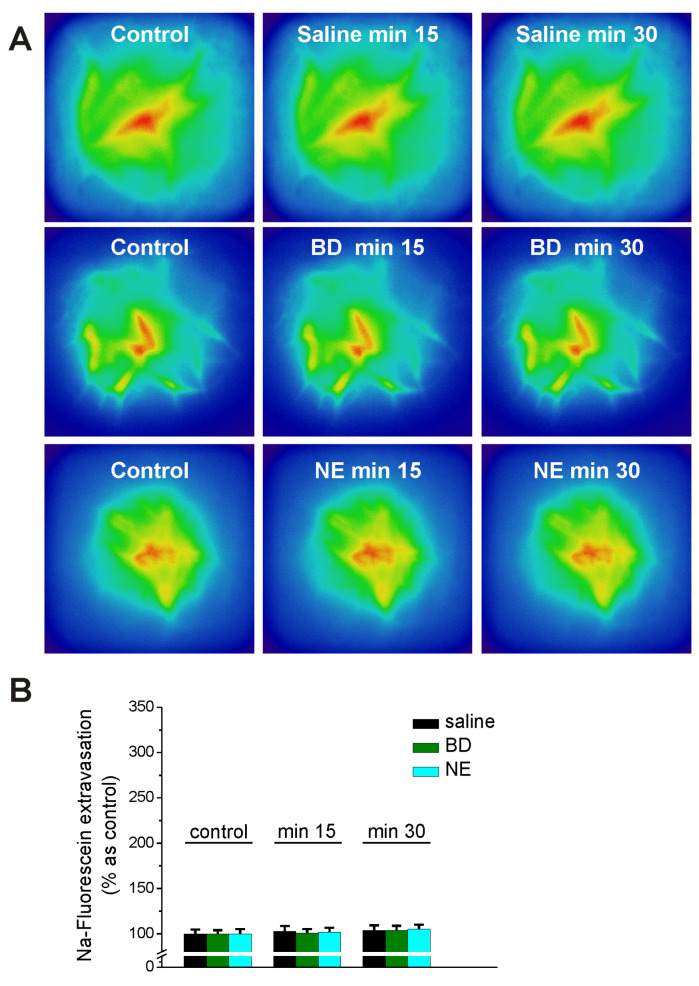
Sigma-1R antagonists, BD 1047 and NE 100, did not increase sodium fluorescein extravasation in rat brain microvessels visualized with a miniscope. (**A**), Pseudocolor images (GRIN lens, Doric Lenses) illustrating Na fluorescein extravasation in rat prefrontal cortex microvessels before (control), 15 min, and 30 min after injection of saline (top panels), BD1047 (10 mg/kg, ip, middle panels) and NE 100 (1 mg/kg, ip, bottom panels). (**B**), Comparison of Na fluorescein extravasation in brain microvessels in each condition. n = 5 rats/treatment group.

Systemic administration of the Sigma-1R antagonists alone BD 1047 (10 mg/kg, ip) and NE 100 (1 mg/kg, ip) did not elicit an increase in Na fluorescein extravasation different from the vehicle (saline) at 15 min and 30 min after administration (Figure 7).

## 3. Discussion

Sigma-1R is an intracellular receptor located on the ER at the mitochondria-associated ER membrane (MAM) that acts as an inter-organelle signaling modulator [2]. MAM plays important signaling roles in Ca^2+^ transfer from the ER to mitochondria, energy exchange, lipid synthesis, and protein folding [37,38]. Upon agonist stimulation, Sigma-1Rs dissociate from BiP to chaperone IP_3_Rs and promote Ca^2+^ transfer from ER into mitochondria [39]. Mitochondrial dysfunction in the context of Sigma-1R activation is a common mechanism in several neurodegenerative diseases such as Alzheimer’s disease, Parkinson’s disease, Huntington’s disease, and amyotrophic lateral sclerosis [37,40]. A characteristic of these neurological disorders is the dysfunction of the blood–brain barrier (BBB), a functional and structural barrier essential for the maintenance and regulation of the neural microenvironment [41].

Cocaine binds to Sigma-1R [23] and has been reported to impair the BBB function [24,25,27]. Using direct visualization of brain microvessels with miniaturized fluorescence microscopy (miniscope) in awake rats, we found that cocaine increased the BBB permeability [28]. In a mouse model of Alzheimer’s disease, induced by intracerebroventricular (icv) injection of Aβ1-42, daily administration of PRE-084 (1 mg/kg) for 21 days attenuated the amyloid-induced BBB disruption by inhibiting amyloid deposition and increasing expression of LRP-1 [29]. In a mouse model of brain ischemia-reperfusion, daily administration of PRE-084 (1 mg/kg) for 7 days reduced the BBB leakage evaluated with Evans Blue [22]. These studies evaluated the chronic effect of Sigma-1R agonists in a mouse model with a compromised BBB function.

Using rat brain microvascular endothelial cells (RBMVEC), an essential component of the BBB that expresses Sigma-1R [9,22], we first tested the effect of the Sigma-1R agonist PRE-084 on mitochondrial Ca^2+^. PRE-084 (1–20 µM), in concentrations similar to those reported before [42], produced a concentration-dependent increase in mitochondrial Ca^2+^ that was abolished by the Sigma-1R antagonists, BD 1047 and NE 100. Similarly, cocaine increases mitochondrial Ca^2+^ in H9C2 cells and rat cardiac myocytes [43].

The release of Ca^2+^ from the ER into the mitochondrial matrix can affect mitochondrial functions such as activation of metabolic enzymes for ATP production and increase reactive oxygen species (ROS) [44]. Previous studies indicate that Sigma-1R agonists increased mitochondrial ROS in mouse brain mitochondria [42] and rat cardiac cells [43,45]. Similarly, in RBMVEC, PRE-084 increased mitochondrial superoxide production in a concentration-dependent manner.

Since an increase in mitochondrial ROS can lead to cytosolic ROS increase [44], we examined the effect of PRE-084 in RBMVEC loaded with CM-H_2_-DCFDA, a fluorescent dye indicator for cytosolic ROS. PRE-084 increased cytosolic ROS in RBMVEC, similar to previous reports in other cell types [43]. The increase in cytosolic ROS produced by PRE-084 was prevented by Sigma-1R antagonists BD 1047 and NE 100.

Mitochondrial oxidative stress of brain endothelial cells has been linked to BBB disruption [46]. To assess the barrier function in vitro, we determined the effect of Sigma-1R activation on the electrical resistance of the RBMVEC monolayer using ECIS, a method that allows the monitoring of resistance over longer periods of time, while maintaining cells in stable conditions [47,48]. PRE-084 reduced the normalized resistance of the RBMVEC monolayer in a dose-dependent manner suggesting an increase in the paracellular permeability.

Microvascular endothelial cells of the BBB form a tight layer, connected via tight and adherens junctions [41]. Tight junctions consist of a network of transmembrane proteins including occludin, claudin-5, and junctional adhesion molecules that connect to the actin cytoskeleton via adaptor proteins, such as ZO-1 [41]. Adherens junctions such as VE-cadherin connect to the cytoskeleton through catenin proteins. Our immunocytochemistry studies indicate that treatment of RBMVEC with PRE-084 produced a disruption in ZO-1 and VE-cadherin immunostaining, F-actin stress fibers, and the formation of intercellular gaps. These morphological changes support the functional studies indicating a decrease in RBMVEC electrical resistance measured with ECIS. Similar changes were produced in brain microvascular endothelial cells by activation of GPR55 [35] and FFA1 receptors [49], or by agonists such as thrombin [33,47] and platelet-activating factor (PAF) [36].

We then examined the in vivo significance on BBB permeability in rats of our in vitro findings. We used classical methods of BBB permeability investigation using Evans Blue and sodium fluorescein. Despite their low molecular weight (LMW), Evans Blue (961 Da) and sodium fluorescein (376 Da), when injected in circulation, have distinct behaviors and patterns of distribution. Evans Blue binds with serum albumin (69,000 Da) and becomes a high molecular weight protein tracer (HMW), while sodium fluorescein remains unbound in LMW form [34]. PRE-084 produced a dose-dependent increase in both Evans Blue and sodium fluorescein in brain extravasation, indicating an increase in BBB permeability.

To directly visualize the effect of Sigma-1R activation on BBB permeability in vivo, we used miniature integrated fluorescence microscope (i.e., miniscope) technology. This state-of-the-art technology was developed to assess neuronal function in vivo using fluorescence probes [50]. We optimized the use of a miniscope to assess the changes in BBB permeability in awake, freely moving rats [28,49,51]. Miniscope imaging of brain microcirculation allows the high-resolution, real-time, visualization of fluorescent tracer extravasation at selected regions of interest (ROIs) in awake rats while avoiding potential impairment of BBB by anesthetics [51]. This is in contrast to classical tracer assays which examine dye content in the whole brain ex vivo, generally 2 h after drug administration. Systemic injection of PRE-084 produced an increase in sodium fluorescein extravasation in brain microvessels at 15 and 30 min after injection. Sigma-1R antagonists alone did not produce sodium fluorescence extravasation; however, their systemic administration abolished the sodium fluorescein extravasation produced by PRE-084. We previously reported that acute cocaine administration produced a transient increase in BBB permeability examined with a miniscope [28]. However, while cocaine can bind to Sigma-1R, it has additional mechanisms of action [52].

Using Sigma-1R selective ligands, we examined the direct effect of Sigma-1R activation on BBB permeability in freely moving rats. To our knowledge, this is the first study to provide evidence that systemic administration of the Sigma-1R agonist produced a fast and transient increase in BBB permeability that was abolished by the Sigma-1R antagonists, BD 1047 and NE 100. Taken together, our results indicate that Sigma-1R activation by PRE-084 promotes oxidative stress and barrier disruption in vitro and increases BBB permeability in vivo.

## 4. Materials and Methods

### 4.1. Chemicals and Reagents

Sigma-1R selective agonist, PRE-084 hydrochloride, [30] and Sigma-1R antagonists, BD 1047 hydrobromide and NE 100 hydrochloride [31,53] were purchased from Tocris (Bio-Techne Corporation, Minneapolis, MN, USA). Other chemicals are from Sigma-Aldrich (St. Louis, MO, USA) unless otherwise noted. PRE-084, BD 1047, and NE 100 solutions were prepared in saline which served as the vehicle control.

### 4.2. Animals

Adult male Sprague Dawley rats (Charles River Laboratories Inc., Wilmington, MA, USA) were housed on a 12 h light/dark cycle with free access to chow and water. Animal protocols were approved by the Institutional Animal Care and Use Committee of Temple University.

### 4.3. Cell Culture

Rat brain microvascular endothelial cells (RBMVEC) from Cell Applications, Inc. (San Diego, CA, USA) were cultured in rat brain endothelial basal medium enriched with endothelial growth supplement, according to the manufacturer’s instructions at 37 °C and 5% CO_2_, as previously reported [35,49]. Cells were grown on T75 flasks coated with an attachment factor (Cell Applications, Inc.). For impedance measurements, cells were grown on an 8W10E+ array (Applied Biophysics, Inc., Troy, NY, USA) coated with fibronectin. For measurements of mitochondrial Ca^2+^, cytosolic ROS, and mitochondrial ROS, RBMVEC were grown on 25 mm diameter glass coverslips coated with fibronectin (50 µg/mL) (Corning, Discovery Labware, Bedford, MA, USA). For immunocytochemistry studies, RBMVEC were grown on 12 mm diameter glass coverslips coated with fibronectin (50 µg/mL).

### 4.4. Mitochondrial Ca^2+^ Measurement

Mitochondrial Ca^2+^ concentration was monitored in RBMVEC loaded with the mitochondrial Ca^2+^ indicator Rhod-2-AM (Invitrogen, ThermoFisher Scientific, Rockford, IL, USA), as previously described [54]. Cells were incubated with 2 µM Rhod-2-AM in Hank’s balanced salt solution (HBSS) at room temperature for 30 min, in the dark, and then incubated for 30 min in HBSS to allow for de-esterification of the dye. Coverslips were mounted in an open bath chamber (RP-40LP, Warner Instruments, Hamden, CT, USA) on the stage of an inverted microscope Nikon Eclipse TiE (Nikon Inc., Melville, NY, USA). The microscope was equipped with a 40× oil immersion objective lens, a Photometics CoolSnap HQ2 CCD camera (Photometrics, Tucson, AZ, USA), and a Perfect Focus System. During the experiments, the Perfect Focus System was activated and Rhod-2-AM fluorescence (excitation/emission-560/582 nm) was acquired at a frequency of 0.1Hz and analyzed using NIS-Elements AR 3.1 software (Nikon Inc.).

### 4.5. Mitochondrial ROS Accumulation

Measurements of mitochondrial ROS levels were carried out using the MitoSOX red, mitochondrial superoxide indicator (Invitrogen), as previously reported [33]. MitoSOX red reagent permeates live cells and rapidly and selectively targets mitochondria. At the mitochondrial level, it is rapidly oxidized by superoxide, and oxidation of the MitoSOX reagent leads to red fluorescence. Cells were incubated with 3 µM MitoSOX red in HBSS at room temperature for 25 min in the dark and washed with dye-free HBSS. The intensity of red fluorescence after excitation at 510 nm was acquired at a frequency of 0.25 Hz and evaluated as a measure of mitochondrial superoxide accumulation.

### 4.6. Cytosolic ROS Accumulation

Cytosolic ROS levels were measured using CM-H_2_-DCFDA [5-6-chloromethyl-27-dichlorodihydrofluorescein diacetate, acetyl ester] (Invitrogen), as previously reported [33]. This assay is based on the principle that CM-H_2_-DCFDA passively diffuses into cells; its acetate groups are cleaved by intracellular esterases and its thiol-reactive chloromethyl group reacts with intracellular glutathione and other thiols. In the presence of ROS, CM-H_2_-DCFDA is rapidly oxidized to become a highly fluorescent product that is trapped inside the cell. Cells were incubated with 1 µM CM-H_2_-DCFDA in HBSS at room temperature for 15 min in the dark and washed with dye-free HBSS. CM-H_2_-DCFDA fluorescence (excitation/emission-495/520 nm), was acquired at a frequency of 0.25 Hz using NIS-Elements AR 3.1 software and monitored as a measure of cytosolic ROS accumulation.

### 4.7. Impedance Measurements

Impedance measurements were performed via the electric cell-substrate impedance sensing (ECIS) method using a Zθ controller, a 16 W array holder station, and gold electrode arrays (8W10E+), as previously reported [35,49]. RBMVEC were cultured at a density of 100,000 cells/cm^2^ on 8W10E+ arrays coated with fibronectin (50 µg/mL, 200 µL/well, 30 min, 37 °C) and treated with L-cysteine (10 mM, 200 µL/well, 30 min, room temperature). Cells were grown on arrays in a complete RBMVEC medium (Cell Applications, Inc.) for 48–72 h in an incubator (37 °C, 5% CO_2_) and then transferred to an FBS-free medium for two hours before drug treatment. Monitoring and acquisition of impedance, resistance, and capacitance were performed using ECIS software v1.2.215 at multiple AC frequencies. To assess the effect of Sigma-1R ligands on barrier function and the paracellular path, the resistance at 4000 Hz frequency averaged for the cells grown on 40 electrodes/well was normalized to the value before the addition of the compound and plotted as a function of time [47,49].

### 4.8. Immunocytochemistry and Fluorescence Microscopy

Immunocytochemistry studies were carried out as previously described [33,35]. RBMVEC grown on coverslips until confluence were treated with PRE-084 (10 µM); untreated cells served as the control. Then, 30 min later, the cells were rinsed in PBS, fixed in 4% paraformaldehyde, washed with PBS, followed by PBS with 0.5% Triton X for 5 min, blocked with normal goat serum, then incubated with primary antibody ZO-1(1:200, rabbit polyclonal, cat # 40–2200, Invitrogen, Thermo Fisher Scientific, Rockford, IL, USA), or VE-Cadherin (1:200, rabbit polyclonal, cat # 36–1900, Invitrogen, Thermo Fisher Scientific) followed by incubation with secondary antibody Alexa Fluor 488 goat anti-rabbit IgG (1:200, cat # A11008, Invitrogen, Thermo Fisher Scientific) for 2 h at room temperature. In another series of experiments, cells were washed in PBS and incubated with ActinRed 555 (Molecular Probes, Eugene, OR, USA) for 30 min, at room temperature, washed in PBS, mounted with DAPI Fluoromount G (Invitrogen, Thermo Fisher Scientific), and sealed. The cells were examined under a Leica DMI6000B fluorescence microscope (Leica Microsystems, Mannheim, Germany) equipped with the appropriate excitation/emission filters.

### 4.9. In Vivo BBB Permeability—Evans Blue Extravasation Method

In vivo assessment of BBB permeability was carried out using the Evans Blue method, as reported earlier [35,49]. Evans Blue (2% in saline; 0.4 mL/kg, i.v. via tail vein) was administered 30 min before the IP injection of Sigma-1R ligands. Two hours later, rats were anesthetized with ketamine (100 mg/kg) and xylazine (5 mg/kg) and perfused transcardially with PBS. After dissection, the brain was weighed and homogenized in PBS, then treated with trichloroacetic acid (80%, 1 h, 4 °C). After centrifugation (10,000 RPM, 20 min), the absorbance (610 nm) of the supernatant was determined using a plate reader along with the brain concentration of Evans Blue.

### 4.10. In Vivo BBB Permeability—Sodium Fluorescein Extravasation Method

BBB permeability was also assessed by measuring the sodium (Na) fluorescein content in the rat brain, as earlier reported [36]. Na fluorescein (2% in saline, 0.5 mL/kg, i.v. via tail vein) was administered 30 min before the IP administration of Sigma-1R ligands. Two hours after drug administration, the rats were anesthetized and transcardially perfused with ice-cold PBS. The brain was removed, rinsed, weighed, and homogenized in PBS. The homogenate was treated with trichloroacetic acid (80%) and centrifuged (10,000 RPM, 20 min). The supernatant was diluted with 5 M NaOH, and the fluorescence was determined (excitation/emission-480/525 nm) using a plate reader. The concentration of Na fluorescein in the brain was quantified and expressed per gram of tissue.

### 4.11. In Vivo BBB Permeability—Miniaturized Fluorescence Microscopy (Miniscope)

Surgical implantation of the imaging cannula (Miniscope GRIN lens, Doric Lenses, Inc., Quebec, QC, Canada) was performed as previously reported [28,51]. Briefly, rats were anesthetized with isoflurane, and the imaging cannula was implanted into the prefrontal cortex using the following stereotaxic coordinates from bregma (AP: 3 mm, ML: 0.5 mm, DV: 2.6 mm). After two weeks of recovery from surgery and habituation to the study procedures, experiments began. Na fluorescein (2% in saline, 0.5 mL/kg) was injected through the tail vein followed 15 min later by PRE-084, BD 1047 + PRE, NE 100 + PRE, vehicle, BD 1047 and NE 100, injected i.p. Na fluorescein extravasation, as an indicator of BBB permeability, was measured immediately prior to (i.e., baseline) and 15 min and 30 min following treatment. Fluorescence intensity (Ex/Em-488/520 nm) was measured in 10 regions of interest (ROIs) in the proximity of microvessels [28,51]. The same 10 ROIs were recorded prior to and following drug administration using a within-subjects design. Fluorescence was visualized, recorded, and analyzed post-acquisition using Doric Studio software version 6.1.5.0 (Doric Lenses, Inc.).

### 4.12. Statistical Analysis

Data are expressed as the mean ± standard of error of the mean (SEM). A one-way ANOVA followed by post hoc analysis using the Bonferroni test was used to evaluate significant differences between groups; a two-sample *t*-test was used when comparing two different groups; *p* < 0.05 was considered statistically significant.

## Data Availability

The data generated and analyzed during this study are available in the manuscript.
